# Deconstructing pathways to resilience: A systematic review of associations between psychosocial mechanisms and transdiagnostic adult mental health outcomes in the context of adverse childhood experiences

**DOI:** 10.1002/cpp.2732

**Published:** 2022-03-18

**Authors:** Corinna Panagou, Angus MacBeth

**Affiliations:** ^1^ School of Health in Social Science University of Edinburgh Edinburgh EH8 9AG UK

**Keywords:** childhood adversity, mediation, moderation, psychological mechanisms, systematic review

## Abstract

Adverse childhood experiences (ACEs) are identified with increased risk of adult mental health difficulties and negative impacts on well‐being. However, there is a need to go beyond simple associations and identify candidate mechanisms underpinning the ACEs–mental health relationship. Further methodological heterogeneity points to issues around the operationalization of ACEs and the importance of modelling data using robust research designs. The aim of the current review was to synthesize studies that utilized formal mediation and/or moderation analyses to explore psychological and social variables on the pathway between clearly defined ACEs (as measured by the ACE questionnaire and Childhood Trauma Questionnaire [CTQ]) and common mental health outcomes (depressive, anxiety and post‐traumatic stress disorder [PTSD] symptoms) across community samples aged over 18. A total of 31 papers were retrieved for critical appraisal. The majority of the studies explored factors mediating/moderating the link between child adversity and depression and less on anxiety and trauma. Most mechanisms were tested in only single studies, limiting the consistency of evidence. Evidence indicated that the mechanisms underlying associations between ACEs and adult mental health are likely to reflect multiple intervening variables. Further, there are substantial methodological limitations in the extant literature including the proliferation of causal inferences from cross‐sectional designs and both measurement and conceptual issues in operationalizing adversity. Consistent transdiagnostic mechanisms relevant to common mental health problems were identified, including perceived social support, emotion regulation and negative cognitive appraisals/beliefs. Further research using longitudinal design is required to delineate the potential contribution of the identified mechanisms.

**Key Practitioner Message**

- There is a need for early identification and implementation of primary, secondary and tertiary interventions with a view to targeting adversity at various levels.
- There is evidence for key mediating processes, such as social support, emotion regulation and cognitive processes, coping strategies, self‐esteem and attachment, that could be taken into account in screening procedures, assessment and targeting of clinical or even preventative interventions.
- Encouraging social inclusion and support via group‐based interventions could help improve symptoms of affective disorders in people exposed to child adversity.
- Therapists working with sufferers of abuse in the past could assess emotion regulation deficits and facilitate the development of emotion regulation skills, if needed, during treatment.

## 1 INTRODUCTION

Exposure to childhood adversity and its associated consequences constitutes a global problem (e.g., Finkelhor et al., 2015; Kessler et al., 2010; Lewis et al., 2019; Manyema & Richter, 2019). Key research and epidemiological data suggest that childhood adversity is a growing public health concern with high prevalence rates (38%–29%) worldwide (Giano et al., 2020; Kessler et al., 2010; Nelson et al., 2020). Since the mid‐1990s, research into adverse childhood experiences (ACEs) (Felitti et al., 1998) has demonstrated that exposure to ACEs may put children and adolescents at higher risk for development of myriad mental health difficulties, such as behavioural problems (McLaughlin et al., 2012), depression (Tsehay et al., 2020), anxiety (Elmore & Crouch, 2020), post‐traumatic stress disorder (PTSD) (Alisic et al., 2014), substance misuse (Carliner et al., 2016) and suicidal ideation (Björkenstam et al., 2017). Childhood adversity is also a risk factor for the development of psychopathology in adulthood (Herzog & Schmahl, 2018), with a substantial body of evidence demonstrating that adults with ACEs are at greater risk for developing a broad range of mental health difficulties, such as anxiety and depressive disorders (Chapman et al., 2004; Danese et al., 2009; Mersky et al., 2013), PTSD (Green et al., 2010; Schoedl et al., 2010), substance misuse (Dube et al., 2003), psychosis (Varese et al., 2012), self‐harm and suicidal ideation (Dube et al., 2003; Thompson et al., 2019).

However, a key conceptual issue in the current literature on ACEs is how researchers conceptualize and measure childhood adversity. The definition of ACEs is relatively broad in that ‘adversity’ is used interchangeably with other concepts, such as ‘trauma’, ‘traumatic experience’, ‘maltreatment’ or ‘stress’. The lack of clarity in the way ‘adversity’ is defined remains an important gap in the literature, spurring attempts to generate greater consistency in the operationalization of the term (McLaughlin, 2016). In addition, there are measurement challenges, with multiple questionnaires used for assessing ACEs (Zarse et al., 2019). For example, Aafjes‐van Doorn et al. (2020) identified 127 questionnaires that have been used in ACEs research. Given the dose–response association between history of childhood adversity and the likelihood of later psychopathology (Dong et al., 2004; Merrick et al., 2017), methods of assessment that provide a cumulative score have been recommended (Bethell et al., 2017; Evans, Li, & Whipple, 2013). However, methodological and conceptual limitations of an additive ACE score remain (e.g., not taking into account the heterogeneity of adverse experiences) (Barboza, 2018).

Importantly, although ACEs have been conceptualized as a risk factor for psychopathology, there is no one to one mapping of ACEs to psychiatric disorder, ergo not everyone who has experienced childhood adversity will experience mental health problems (Belsky & Pluess, 2009). Evidence suggests the impact of ACEs may depend on various factors, such as the type of event and severity of exposure (Schalinski et al., 2016), the doseage or cumulative effects of adverse events (Edwards et al., 2003), age of exposure (Dunn et al., 2013; Riem et al., 2015, and the socio‐economic context (Nurius et al., 2012). This suggests a need to understand the multi‐factorial nature of ACEs and unpack mechanisms by which ACEs are implicated in potential trajectories of risk (psychopathology) and resilience.

Indeed, when exploring trajectories for psychopathology risk, recent transdiagnostic models have identified multiple shared underlying processes across various disorders, including genetic factors (e.g., Selzam et al., 2018), socio‐environmental variables, such as poverty, family functioning and child maltreatment (Farah et al., 2006; Vachon et al., 2015), and psychological factors, such as emotion processing (McLaughlin, 2016). Furthermore, resilience‐focussed research with youths and adults has shown that positive adaptation in the aftermath of adversity emerges from a dynamic interplay of various factors, including cognitive processes, coping strategies, individual traits, timing of the traumatic event, social context and support and relationship with caregivers (Masten, 2011; Rutter, 2006).

Transdiagnostic models also provide a novel framework for conceptualizing mental health problems accounting for multiple aspects, such as complexity, multifinality and comorbidity. Applying multifinality—by which a single risk factor can lead to a broad range of difficulties—to mental health has generated models of psychopathology that identify core and shared underlying processes across different types of psychopathology (Dalgleish et al., 2020; Nolen‐Hoeksema & Watkins, 2011). There is also evidence that childhood adversity is associated with high prevalence of depression, anxiety and posttraumatic stress (e.g., Herzog & Schmahl, 2018; Lee et al., 2020; van der Feltz‐Cornelis et al., 2019), while it has been argued that in the context of ACEs, these disorders may cluster together as a discrete group (Teicher & Samson, 2013). Similarly, an increasing body of research indicates comorbidity across depression, anxiety and PTSD (Forbes et al., 2011) which implies potentially co‐occurring commonalities among these discrete disorders. Therefore, these findings highlight the need for further evidence as to how ACEs are related common mental health difficulties and their underlying mechanisms.

Despite the well‐established link between ACEs and mental health, research exploring the mediational processes through which adversity leads to risk has been historically underdeveloped. However, over the last 10 years, there has been a growth in studies using formal mediation and moderation analysis techniques to explore the role of biological and psychosocial mechanisms in clinical and general populations. The aim of mediation is to strengthen quasi‐causal inferences regarding the mechanisms through which an independent variable affects an outcome, while the aim of moderation is to examine variables affecting the strength/direction of the predictor–outcome relationship (Hayes & Preacher, 2010; MacKinnon, 2011). Therefore, mediation and moderation testing can help identify interdependencies between early adversity and mental health and explore the role of processes that lie between early adversity and mental health later in life. Importantly, examining third‐variable effects is consistent with moving beyond associative relationships between variables towards identifying risk and protective factors and thus creating more stratified and nuanced models for mental health phenomenology and intervention.

Previous reviews have explored the link between adversity in childhood and psychopathology, focused on specific forms of adversity or traumatic experiences (e.g., Whiffen & MacIntosh, 2005), various types of psychopathology, including complex psychiatric disorders (e.g., Alameda et al., 2020), and specific mechanisms, such as neurobiological (e.g., McCrory et al., 2012) or cognitive ones (Aafjes‐van Doorn et al., 2020), and included studies utilizing clinical or/and community samples. Hoppen and Chalder (2018) aimed to synthesize and critically evaluate the role of biological and psychosocial mediators and moderators on the link between child adversity and affective disorders in adults, including papers up to October 2017. This review was characterized reportedly by heterogeneity and utilized broad inclusion criteria, e.g., wide range of questionnaires measuring adversity, a variety of samples, such as clinical, non‐clinical and specific populations, and inclusion of correlational as well as mediation and moderation studies, the latter forming a minority of the reviewed studies.

The current review sought to increase specificity through the adoption of narrower eligibility criteria, correspondingly reducing methodological variance. Specifically, to address the disparity that characterizes the literature base due to the operationalization and measurement of child adversity and improve specificity and validity, studies were only included if they measured child adversity via the ACE questionnaire (Felitti et al., 1998) or Childhood Trauma Questionnaire (CTQ) (Bernstein et al., 2003), which are the most widely used retrospective questionnaires of adversity in childhood that give a cumulative score of ACEs (Schmidt et al., 2020; Tonmyr et al., 2011; Zarse et al., 2019). Both questionnaires have been utilized extensively in research and were shown to have good psychometric properties (Bernstein et al., 2003; Dube et al., 2003). Furthermore, the search focused on three categories of mental health outcomes on the grounds that there is a high rate of comorbidity and common underlying psychological processes linked with anxiety, depression and PTSD in the context of ACEs (e.g., Purdon, 1999; Renna et al., 2017; Watkins, 2015). General population studies were included to overcome the effects of further confounding variables, prevent data skew and allow for generalisability of findings to the community. Finally, studies were only included if they used formal approaches to mediation analyses, such as product of coefficient, difference in coefficient, Baron and Kenny approach, confirmatory test of complete mediation, and SEM or significance tests of mediation, such as Baron and Kenny's Causal‐Steps approach (Baron & Kenny, 1986), joint significant test (Mackinnon et al., 2002), the Sobel first‐order test (Sobel, 1982), PRODCLIN (Mackinnon et al., 2007) and bootstrapping (Hayes, 2017; Johnson, 2001).

Therefore, the objective of the present review was to identify, synthesize and systematically evaluate studies that utilized formal mediation and/or moderation analyses to measure the effect of psychological variables upon the association between clearly defined ACEs (as measured by the ACE questionnaire and CTQ) and common mental health outcomes (depression, anxiety and PTSD symptoms) across general population samples.

## 2 METHODS

### 2.1 Literature search strategy

A narrative systematic review was carried out in accordance with the Preferred Reporting Items for Systematic Reviews and Meta‐Analyses (PRISMA) guidelines (Moher et al., 2015). A systematic search of English language articles published from inception to September 2020 was conducted utilizing Medline, PsychINFO, Embase, CINAHL and ERIC. Google Scholar was also conducted to identify non‐indexed papers. Identification of studies through manual review of reference lists of existing reviews in the field and retrieved articles was used to identify additional relevant articles. Keywords searched were related to the following three sets of terms combined with the Boolean operator ‘and’: (1) ‘child adversity’, (2) ‘mediation’ and ‘moderation’ and (3) terms representing mental health outcomes (‘anxiety’, ‘depression’ and ‘PTSD/trauma symptoms’; see Appendix S1 for complete search strategy).

In the current literature, ‘child adversity’ is often used interchangeably with a wide range of terms (e.g., trauma and maltreatment) which were utilized as keywords to include a broad range of operational definitions of the term. Regarding the second set of key terms, both mediation and moderation analysis can help explore relationships between variables. First, mediation analysis is herein defined as the process that examines the effect of an intervening variable on the relationship between a predictor and an outcome variable (Fairchild & McDaniel, 2017). While several methods have been designed to investigate indirect effects between variables, there is not a unified approach in mediation with an agreed set of assumptions (Hayes, 2017). Second, moderation analysis is defined as the process that tests whether the prediction of the outcome from an independent variable is different across different levels of a third variable (i.e., the moderator) (Fairchild & MacKinnon, 2009; Hayes, 2017).

Finally, the third set of key terms included three categories of mental health outcomes: depression, anxiety and PTSD or trauma symptoms. Studies were included if they used relevant mental health outcome measures.

### 2.2 Eligibility criteria

Quantitative empirical studies were included in the review, if they (1) were written in English language and published as original research in a peer‐reviewed journal, (2) used a cross‐sectional or longitudinal research design, (3) reported exposure to ACEs before the age of 18, utilizing either the ACE questionnaire and adaptations (the original 7 item or its 10 item expanded form) or the CTQ questionnaire and its adaptations, (4) measured psychosocial (i.e., related to affect, behaviour, cognitions or mood as well as social and interpersonal processes) mediating and/or moderating factors, (5) used outcome measures for depression, anxiety and trauma/PTSD symptoms, (6) used formal methods for testing mediated or indirect effects (e.g., such as bootstrapping or SEM), and (7) used a general population sample aged over 18 years of age. There were no restrictions on study settings.

Studies were exclude if they (1) were case reports, reviews, open trials, dissertations, conference abstracts or qualitative studies, (2) used specific populations (e.g., clinical samples, veterans, offenders, etc.), and (3) utilized general mental health outcome questionnaires.

### 2.3 Selection process and data extraction

After duplicates were removed, titles and abstracts obtained were reviewed against eligibility criteria. Full texts of studies meeting the search criteria were retrieved for further screening. Uncertainty regarding article eligibility was resolved through consensus discussion with the second author (AM). Data from studies meeting the inclusion criteria were extracted into a data extraction form to categorize relevant information from selected research articles (Tables 1 and 2). Information was extracted from each individual study on (1) participant characteristics and sample size; (2) study design; (3) measurement of childhood adversity; (4) psychological and social mediating and moderating variables; (5) mental health outcome; (6) type of analysis; and (7) key research findings.

**TABLE 1:** Study characteristics and key research findings analyses (community samples)

Study (author, year; country)	Population	Sample size (*N*); female %; mean age	Study design	Measurement of child adversity; definition of childhood adversity	Psychosocial mediating variables; measurement	Psychosocial moderating variables; measurement	Mental health outcomes; measurement	Type of mediation analysis; potential confounders or covariates considered (yes/no)	Mediation/moderation results/key findings (pathway total/partial mediation, direct effect [DE], indirect effect [IE], % total effect mediated)
Cantave et al. (2019), Canada	Community sample part of project aiming to explore the biosocial roots of aggression.	156; 0%; 24.10	Cross‐sectional	CTQ‐SF; child maltreatment	Task‐oriented, emotion‐oriented and avoidance‐oriented coping strategies; the Coping Inventory of Stressful Situations (CISS; Endler & Parker, 1994)	Task‐oriented, emotion‐oriented & avoidance‐oriented coping strategies; the Coping Inventory of Stressful Situations (CISS; Endler & Parker, 1994)	Depression; Beck Depression Inventory‐II (BDI‐II; Beck et al., 1996)	Bootstrapping (BC; 95% CI), PROCESS Macro (Hayes, 2013);No	Significant IE of maltreatment on depression via emotion‐oriented coping strategies (*b* = 1.02 [*SE* = 0.50], BC 95% CI [0.24–2.20]). IE accounted for 20% of the total effect. Partial mediation.No IE found for task‐oriented and avoidance‐oriented coping strategies.Avoidance‐oriented and emotion‐oriented coping strategies did not moderate the maltreatment‐depression relationship.Task‐oriented coping strategies moderated the maltreatment‐depressive symptoms association (*r* ^2^ = 0.02, *b* = −0.23, *t*(148) = 2.08, *p* = 0.04]. 0.04].
Crow et al. (2014), USA	Community sample recruited from the general medical gynaecological clinics	3,902; 68,9%; 30.34	Cross‐sectional	CTQ‐SF;Childhood trauma	Emotion dysregulation; Emotion Dysregulation Scale, short version (EDS; Powers et al., 2015)	None tested	Depression; Beck Depression Inventory‐II (BDI‐II; Beck et al., 1996)	Bootstrapping (BC; 95% CI), PROCESS Macro (Hayes, 2008);No	Significant IE (*a x b =* 0.525, 95% CI [0.48–0.55]) of childhood emotional abuse on depression through emotion dysregulation.Emotional abuse stronger predictor of adult depression.
Evans, Steel, and DiLillo (2013), USA	Community sample recruited from marriage licence records.	372; 50%; 26.59	Cross‐sectional	CTQ; child maltreatment	None tested	Social support;Perceived Social Support Index (PSS; Procidano & Heller, 1983)	Trauma symptoms;Trauma Symptom Inventory (TSI; Briere, 1995)	Moderation analysis. Two‐step hierarchical regression model, Baron and Kenny (1986) approach;No	Perceived social support (PSS) from family predicted moderation of the relationship between physical abuse (*R* ^2^ = 0.09, *p* = 0.007), emotional abuse (*R* ^2^ = 0.13, *p* = 0.009), emotional neglect (*R* ^2^ = 0.12, *p* = 0.008) and trauma.PSS from family did not moderate the child maltreatment‐ trauma symptoms for men. PSS from friends did not moderate the child maltreatment‐trauma link for both men and women.
Fitzgerald and Gallus (2020), USA	Community sample. Data taken from MIDUS study (midlife development in the United States.	798; 49,1%; 57.52	Longitudinal	CTQ; childhood maltreatment	Emotional support from family, friends and romantic partner questionnaires. Items specific to MIDUS study.	None tested	Depression; Centre for Epidemiologic Studies Depression (CES‐D; Radloff, 1977)Social Anxiety; Liebowitz Social Anxiety Scale (LSAS; Fresco et al., 2001)	Multiple mediator model. SEM, path analysis;Yes	DE of childhood maltreatment on depression (*ß* = 0.22, *p* < 0.05) and social anxiety (*ß* = 0.10, *p* < 0.05).Significant IE of child maltreatment on depression via emotional support from family (*b* = 0.09; 95% CI [0.045–0.146]) and romantic partner (*b* = 0.02; 95% CI [0.005–0.048]) but not friends (*b* = 0.02; 95% CI [−.002 to 0.040]). 20.9% of variance in depression.Significant IE of child maltreatment on social anxiety through emotional support from friends (*b* = 0.04; 95% CI [0.019–0.066]) and romantic partners (*b* = 0.03; 95% CI [0.008–0.048]). 7.3% of variance in social anxiety of variance in social anxiety.
Hayward et al. (2020), USA	Community sample recruited through amazons mechanical Turk.	382; 48.2%; 35.60	Cross‐sectional	CTQ‐SF; childhood adversity	Self‐concept clarity; Self‐Concept Clarity Scale (Campbell et al., 1996)Intolerance of uncertainty; Intolerance of Uncertainty Scale‐12 (IUS‐12; Carleton et al., 2007)	None tested	Depression; Depression subscale of the Depression Anxiety Stress Scale (DASS‐21; Lovibond & Lovibond, 1995)Generalized anxiety;GAD‐7 Questionnaire (Spitzer et al., 2006)OCD symptoms; Obsessive–Compulsive Inventory‐Revised (OCI‐R) scale (Foa et al., 2002)Social anxiety; Social Phobia Inventory (SPIN; Connor et al., 2000)	Parallel and serial mediation models; SEM;No	Significant IE of child adversity through SCC on:Depression (*b* = 0.07; 95% CI [0.05–0.10, *p* = <0.001);GAD (*b* = 0.04; 95% CI [0.02–0.05, *p* = <0.001);OCD C (*b* = 0.09; 95% CI [0.05–0.10], *p* = <0.001);Social anxiety (*b* = 0.13; 95% CI [0.07–0.19, *p* = <0.001).Both SCC and IOU mediated the link between child adversity and mental health outcomes.54% of the variance in depression, 55% in GAD, 44% in OCD, and 54% in social anxiety.In the model child adversity: Childhood trauma (0.94) and risk families (0.90). 2 IV.
Gomes Jardim et al. (2019), Brazil	Community sample‐ elderly population.	260; 76.9%; 72.2	Cross‐sectional	CTQ‐SF; childhood maltreatment	Personality factors: NEO‐Five Factor Inventory (NEO‐FFI; Costa and McCrae, 1995)	None tested	Depression; Mini International Neuropsychiatric Interview 5.0 plus Portuguese version (MINI plus) (Amorim, 2000)	SEM;No	Significant IE of childhood maltreatment on depression via neuroticism (*b* = 0.24, SE = 0.040, *p* < 0.001) and extraversion (*b* = 0.09, SE = 0.022, *p* < 0.001). Total mediation effect.Non‐significant effect of openness (*b* = 0.02, *SE* = 0.012, *p* = 0.091). Significant IE of CTQ score on depression via agreeableness (*b* = 0.03, *SE* = 0.017, *p* = 0.047) and conscientiousness (*b* = 0.05, *SE* = 0.016, *p* = 0.005). Partial mediation effect.
Kim et al. (2017), South Korea	Community sample recruited through local newspapers and posters	1,027; 68.6%; 45.1	Cross‐sectional	K‐CTQ (Korean version); childhood maltreatment	Rumination;Ruminative Response Scale (RRS; Nolen‐Hoeksema, 1991)	None tested	Depression; Beck Depression Inventory‐II (BDI‐II; Beck et al., 1996)Anxiety;SAI subscale of State Anxiety Inventory (SAI; Spielberger et al., 1983)	SEM; bootstrapping. Sobel test;Yes	Significant DE (*b* = 0.29, CI [0.003–0.49], *p* = 0.01) and IE (*b* = 0.38, CI [0.25–0.56], *p* = 0.01) of childhood maltreatment on mood through rumination.Total effect: 0.67IE: 0.38Significant mediating role of rumination (Sobel test: 4.19).
Klumparendt et al. (2019), Germany	Community sample recruited via PsyWeb.	1,027; 68.6%; 45.1	Cross‐sectional	CTQ‐SF (German version); childhood maltreatment	Emotion regulation;Difficulties in Emotion Regulation Scale (DERS; Gratz & Roemer, 2004). German versionAttachment;Experiences in Close Relationship Scale (ECR; Brennan et al., 1998). German versionAttributional style;Depressive Attributions Questionnaire (DAQ; Kleim, et al., 2011). German versionPTSD;PTSD‐Checklist for DSM‐5 (PCL‐5; Weathers et al., 2017)	None tested	Depression; Patient Health Questionnaire (PHQ‐8; Kroenke, Spitzer & Williams, 2001)German version	Multiple mediator model. SEM;No	Significant total IE of childhood maltreatment on depression through mediators (*b* = 0.32; *p* < 0.001) and not significant DE of CM on depression in total model (*b* = 0.002, *p* = 0.957). Total mediation.Emotion regulation, depressogenic attributional style and PTSD symptom severity mediated the relationship between childhood maltreatment and depression.Emotion regulation: strongest IE of limited access to emotion regulation strategies (*b* = 0.09, 95% CI [0.042–0.132], *p* < 0.001) and lack of emotional clarity (*b* = 0.04, 95% CI [0.017–0.067], *p* < 0.01).Significant IE through PTSD (*b* = 0.11, 95% CI [0.72–0.137], *p* < 0.001).Significant IE through depressogenic attribution style (*b* = 0.04, 95% CI [0.11–0.72], *p* = <0.01).No IE for attachment anxiety or avoidance
Kogan et al. (2021), USA	Community sample recruited from rural counties. Data from the African American Mens project.	505; 0%; 21.85 (T1 collection) and 23.12 (T2 collection	Longitudinal	ACE questionnaire;Childhood maltreatment	Defensive schemas; Street Code measure (Stewart & Simons, 2006)Experiences in Close Relationships Scale (Wei et al., & Vogel, 2007)Cynical views of relationships (Simons et al., 2012).Social developmental factors measured by Network of Relationships Inventory (Furman & Buhrmester, 1985Multidimensional Measure of Religious Involvement (Levin et al., 1995)Contextual stressors measured by:Schedule of Racist Events (Landrine & Klonoff, 1996),Economic distress (Cho & Kogan, 2016)The community crime (Sampson et al., 1997).	None psychosocial moderator tested.Moderation analysis included OXTR DNAm	Depression 10‐item version of the Center for Epidemiologic Studies Depression Scale (CES‐D; Björgvinsson, et al., 2013)	Path analysis. Mplus. Bootstrapping (BC; 95% CI);No	ACEs predicted young adult contextual stress (*b* = 0.39, *p* < 0.001), which in turn forecast increases in defensive/hostile schemas (*b* = 0.25, *p* < 0.05). Defensive/hostile schemas predicted increases in social developmental risk factors (*b* = 0.15, *p* < 0.05), which were a proximal antecedent of depressive symptomatology (*b* = 0.10, *p* < 0.05) and substance abuse (*b* = 0.24, *p* < 0.001).No DE of ACEs on relational schemas.Concurrent stressors mediated the link between ACEs relational schemas.IE linking contextual factors to social developmental factors via defensive relational schemas only when levels of OCTR methylation were high (*b* = 0.13, 95% CI [0.013–0.242]).OCTR‐DNA methylation = moderator.ACEs affect mental health outcomes indirectly through contextual contemporary factors.
McQuaid et al. (2015), Canada	Community sample of first nations recruited from Aborigincal community/health centres.	225; 75.11%; 35.8	Cross‐sectional	A 20‐item modified version of the CTQ;Childhood trauma	Unsupportive social interactions; Unsupportive Social Interactions Inventory (USII: Ingram et al., 2001)Perceived discrimination; Perceived Ethnic Discrimination Questionnaire (Contrada et al., 2001)	Ingroup and outgroup unsupport;Unsupportive Social Interactions Inventory (USII: Ingram et al., 2001)	Depression; Beck Depression Inventory short form (BDI‐S; Beck & Beck, 1972)	Single mediation model and moderated mediation model.Multiple mediation. Bootstrapping (BC; 95% CI), PROCESS Macro (Hayes, 2008);Yes	IE of childhood trauma on depression through perceived discrimination. Partial mediation. Significant DE of childhood trauma on depression (*b* 0.08, *p* < 0.01).Moderated mediation analyses showed that the intervening role of discrimination was stronger when levels of outgroup unsupport were higher. Ingroup unspport did not moderate the mediated relationship (*p* = 0.30).Multiple mediation analyses: Emotion focused coping mediated the relationship between childhood trauma and depression (95% CI [0.03–0.11]. The path between emotion‐focused coping and depression was moderated by both ingroup and outgroup unsupport.
Mishra and Marceau (2020), USA	Community sample. Data taken from MIDUS (Brim, Ryff, & Kessler, 2004	1,053; 55%; 55.79	Longitudinal	CTQ;Childhood maltreatment	Perceived stress; Perceived Stress Scale (Cohen et al., 1983)	None tested	Depression;the Recent Care of Common Mental Disorder in the United States: Prevalence and Conformance with Evidence‐Based Recommendations (Wang et al., 2000)	Path models. Mplus 7.4;Yes	IE: The effect of sexual abuse on depression partially explained by perceived stress levels in middle life (*b* = 0.04, *p* = 0.012).The effect of physical and emotional maltreatment on depression fully mediated by both perceived stress (*b* = 0.04, *p* = 0.004) (and cortisol).
Stevens et al. (2013), USA	Community sample recruited from an outpatient gynaecological clinic.	139; 100%; 28.46	Cross‐sectional	CTQ;Childhood abuse	Emotion Regulation; The Difficulties With Emotion Regulation Scale (DERS; Gratz & Roemer, 2004)Social support;Social Supports Provision Scale (SSPS; Cutrona & Russell, 1987)Interpersonal violence;Conflict Tactics Scale (CTS; Straus, 1979)	None tested	Ptsd;PTSD Symptom Scale–Self Reported (PSS‐SR; Foa et al. 1993)	Multiple mediation model. Bootstrapping (BC; 95% CI) (Preacher & Hayes, 2008);Yes	Significant IE of childhood trauma on PTSD through emotion regulation (*b* = 0.13, CI 95% [0.07–0.21]).Significant IE of child abuse on PTSD though interpersonal violence & social support.Emotion regulation mediates the relationship between child abuse and trauma. Effect significant after adjusting for social support and interpersonal violence.Social support and interpersonal violence mediates the relationship between child abuse and trauma. The effect of emotion regulation was stronger.
van Assche et al. (2020), Belgium	Community sample. Older adults	81; 64%; 74.90	Cross‐sectional	CTQ;Childhood trauma	Attachment;Experiences in Close Relationships – Revised (ECR‐R; Fraley et al., 2000)	None tested	Anxiety;Anxiety subscale of the Hospital Anxiety and Depression Scale (HADS‐A; Zigmond & Snaith, 1983)Depression;Geriatric Depression Scale (GD; Yesavage et al., 1982)	Bootstrapping (BC; 95% CI) (Preacher & Hayes, 2004);No	Significant IE of emotional neglect on late life anxiety (*b* = 0.4. 95% CI [0.00–0.15], *p* = 0.03)And depression (*b* = 0.05, 95% CI [0.00–0.17], *p* = 0.04) through attachment anxiety.No DE found between emotional neglect and depression or anxiety.
Wong et al. (2019), USA	Community sample recruited via amazons mechanical Turk.	308; 45.12%; 35.49	Cross‐sectional	ACE questionnaireAdverse childhood experiences	Self‐concept clarity;Self‐Concept Clarity (SCC; Campbell et al., 1996)Self‐esteem;Self‐Esteem scale (Rosenberg, 1965)	None tested	Depression;Beck Depression Inventory‐II (BDI‐II; Beck et al., 1996)Perceived stress; Perceived Stress Scale (Cohen, Kamarck, & Mermelstein, 1983).	Bootstrapping (BC; 95% CI) (Hayes, 2013). Mplus 7. Single and dual mediation model (Muthén & Muthén, 1998–2012);Yes	Significant IE of ACEs on depression through self‐concept clarity (SCC) (*b* = 0.10, 95% CI [0.04–0.16] *p* = 0.001, *r* ^2^ = 0.38) and perceived stress (*b* = 0.11, 95% CI [0.04–0.18], *p* = 0.001, *r* ^2^ = 0.41). 27% of variance in depression, 31% of variance in stress.Significant IE of ACEs on depression *(b* = 0.16, 95% CI [0.09–0.23], *κ* ^*2*^ = 0.18)43% of total effect) and perceived stress (*b* = 0.14, *p* = <0.001, 95% CI [0.07–0.20], *κ* ^2^ = 0.15, 39% of total effect) via self‐esteem.Self‐esteem had larger effect sizes compared to self‐concept clarity.

Abbreviations: ACE questionnaire, CDC‐Kaiser Permanente Adverse Childhood Experience Questionnaire; Felitti et al., 1998); CTQ, Childhood Trauma Questionnaire (Bernstein & Fink, 1998; Bernstein et al., 2003); CTQ‐SF, Childhood Trauma Questionnaire short form; DI, direct effect; IE, indirect effect.

**TABLE 2:** Study characteristics and key research findings analyses (student samples)

Study (author, year; country)	Population	Sample size (*N*); female %; mean age	Study design	Measurement of child adversity; definition of childhood adversity	Psychosocial mediating variable(s); measurement	Psychosocial moderating variable(s); measurement	Mental health outcomes; measurement	Type of mediation analysis; potential confounders or covariates considered (yes/no)	Mediation/moderation results/key findings (pathway total/partial mediation, direct effect [DE], indirect effect [IE], % total effect mediated)
Berman et al. (2019), USA	Female college students	252; 100%; 19.2	Cross‐sectional	ACE questionnaire; Child maltreatment	Negative beliefs; Posttraumatic Maladaptive Beliefs Scale (PMBS; Vogt, Shipherd & Resick, 2012)	None tested	Depression; Patient Health Questionnaire (PHQ‐9; Spitzer et al., 1999 Anxiety; Patient Health Questionnaire (PHQ; Spitzer et al., 1999) Posttraumatic symptoms; Posttraumatic Checklist for DSM‐5 (PCL‐5; Weathers et al., 2013)	Structural equation modelling (SEM). Bootstrapping; Yes	Significant indirect effect (IE) of child maltreatment (abuse and neglect subscales) (*b* = −0.09, *p* = 0.005) on internalizing symptoms (depression, anxiety and posttraumatic symptoms) via negative core beliefs. Partial mediation. Significant IE of household dysfunction (abuse and neglect subscales) (*b* = −0.11, *p* = 0.019) on internalizing symptoms via negative core beliefs. Complete mediation.
Brown et al. (2016), USA	Undergraduate students recruited from a public University in the Midwest United States.	339; 48.7%; 19	Cross‐sectional	CTQ‐SF; Child maltreatment	Alexithymia; Toronto Alexithymia Scale (TAS‐20; Bagby et al., 1994).	None tested	Depression; Short Mood and Feelings Questionnaire (SMFQ; Sharp et al., 2006) Anxiety; General Anxiety Disorder Scale (GAD‐7; Spitzer et al., 2006)	Bootstrapping; No	IE of emotional neglect on depression (*b* = 0.04, 95% CI = [0.003–0.07]) and anxiety (*b* = 0.07, BC 95% CI = [0.01–0.13]) via alexithymia.
Browne and Winkelman (2007), Australia	Psychology undergraduate students recruited from an Australian university.	219; 81.74%; 20.96	Cross‐sectional	CTQ‐SF; Childhood trauma	Adult attachment; Relationship Scales Questionnaire (RSQ; Griffin & Bartholomew, 1994) Cognitive distortions; Cognitive Distortions Scale (Briere, 2000)	None tested	Trauma symptoms; Trauma Symptom Inventory (Briere, 1995)	Structural equation modelling (SEM). Path analysis. Multiple mediation model; No	IE (=0.23) of childhood trauma on trauma symptoms via cognitive distortions. Associations found between childhood trauma and attachment dimensions which on their own were not significantly associated with trauma symptoms.
Corcoran and McNulty (2018), Ireland	University students recruited from Republic of Ireland universities.	190; 76.32%; 22.02	Cross‐sectional	ACE questionnaire; Adverse childhood experiences	Attachment; Experiences in close relationships − relationship structures scale (ECR‐RS; Fraley et al., 2011)	None tested	Depression‐Anxiety‐Stress; Depression anxiety & stress scales (DASS‐21; Lovibond & Lovibond, 1995)	Multiple mediation model. Bootstrapping (BC; 95% CI), PROCESS Macro (Preacher & Hayess (2004); No	Significant IE of childhood adversity on depression through attachment anxiety‐general (*b* = 0.33, BC 95% CI [0.19–0.53]), attachment anxiety‐friend (*b* = 0.09, BC 95% CI [0.01–0.23]) and attachment avoidance‐mother (*b* = 0.21, BC 95% CI [0.06–0.42]). Complete mediation. Significant IE of childhood adversity on anxiety via attachment anxiety‐general (*b* = 0.28, BC 95% CI [0.15–0.46]). Complete mediation.
Gong and Chan (2018), China	College students recruited from universities in China.	1,102; 73.14%; 20.46	Cross‐sectional	CTQ‐SF; Childhood maltreatment	Early maladaptive schemas; Young Schema Questionnaire, version 3 (YSQ‐3; Young et al., 2003). Chinese version	None tested	Depression; Zung Self‐Rating Depression Scale (SDS; (Zung, 1986) Anxiety; Zung Self‐Rating Anxiety Scale (SAS; Zung, 1971)	SEM. Bootstrapping (BC; 95% CI), PROCESS Macro (Preacher & Hayess (2004); No	Significant IEs of physical abuse (BC 95% CI [0.142–0.260]), emotional abuse (BC 95% CI [0.205–0.314]), and sexual abuse (BC 95% CI [0.069–0.176 3]) on psychological distress (depression and anxiety) through early maladaptive schemas. IEs of physical neglect (BC 95% CI [0.142–0.260]) and emotional neglect (BC 95% CI [0.177–0.282]) on depression and anxiety through early maladaptive schemas.
Kaloeti et al. (2019), Indonesia	Undergraduate students recruited from a University in Indonesia.	443; 73.45%; 18.60	Cross‐sectional	ACE questionnaire; Adverse childhood experiences	Resilience; Connor‐Davidson Resilience Scale (CD‐RISC; Connor & Davidson, 2003)	None tested	Depression; Beck Depression Inventory‐II (BDI‐II; Beck et al., 1996)	Path analysis	No DE of ACEs on resilience identified (*b* = 0.37, *SE* = 0.437, *β* = 0.04). Resilience did not mediate the ACEs‐depression relationship.
Makriyianis et al. (2019), USA	College students recruited from a public arts college in US.	305; 66.6%; 19.13	Cross‐sectional	ACE questionnaire; Adverse childhood experiences	Psychological Flexibility and Inflexibility; The Multidimensional Psychological Flexibility Inventory (MPFI; Rolffs et al., 2016)	None tested	Depression; Patient Health Questionnaire (PHQ‐8; Kroenke et al., 2009) Anxiety; General Anxiety Disorder Scale (GAD‐7; Spitzer et al., 2006)	Bootstrapping (BC; 95% CI), PROCESS Macro (Hayes, 2018); Νο	Significant IE of ACEs on depression via inflexibility (*b* = 0.14, *SE* = 0.03, BC 95% CI [0.07–0.21]). 53% of variance in depression. Complete mediation. Significant IE of ACEs on anxiety via inflexibility (*b* = 0.13, *SE* = 0.03, BC 95% CI [0.07–0.20]). 49.2% of the variance. Complete mediation.
McCormick et al. (2017), USA	Students recruited from a south‐eastern university in US.	458; 61.3%; 20.70	Cross‐sectional	ACE questionnaire; Adverse childhood experiences	Religious/spiritual struggles; Religious and Spiritual Struggles Scale (RSSS; Exline et al., 2014)	None tested	Depression; Patient Health Questionnaire (PHQ‐8; Kroenke et al., 2001) PTSQ; Posttraumatic Stress Disorder Checklist‐Civilian Version (PCL‐C; Weathers et al., 1993)	Bootstrapping (BC; 95% CI), PROCESS macro. Sobel test; Yes (age, gender, ethnicity, traumatic life events)	Significant IE of ACEs on depressive symptoms through the meaning struggles (*b* = 0.154, BC 95% CI [0.059–0.291], z = 3.05, *p* = 0.002). Significant IE of ACEs on PTSD symptoms through the meaning struggles subscale of the RSSS (*b* = 0.35, BC 95% CI [0.138, 0.7], z = 3.024, *p* = 0.003).
Nowalis et al. (2020), USA	Students recruited from a north‐eastern university in US.	203; 52%; 19.85	Cross‐sectional	CTQ‐SF; Bernstein et al., 2003 Child maltreatment	None tested	Attachment; Experiences in Close Relationships–Revised (ECRR; Fraley et al., 2000)	Depression; Beck Depression Inventory–II (BDI‐II; Beck et al., 1996).	Path analysis in MPlus; Yes	Anxious attachment to primary caregiver significantly moderated the child maltreatment‐depression relationship (*b* = −0.16 *p* = 0.002, BC 95% CI = [−0.25 to −0.06]). 43.5% of variance. No significant effect identified for avoidant attachment to primary caregiver and for anxious or avoidant attachment to secondary caregiver.
Reiser et al. (2014), Canada	University students recruited through an undergraduate research pool website.	264; 81.06%; 22.56	Cross‐sectional	ACE questionnaire; Adverse childhood experiences	None tested	Negative affect; Positive and Negative Affect Schedule‐Negative Affect (PANAS‐NA) subscale; Watson, Clark, & Tellegen, 1988) Trait Anxiety; Trait form of the State–Trait Anxiety Inventory for adults‐form Y (STAI‐T; (Spielberger et al., 1983)	Health anxiety Short Health Anxiety Index (SHAI; (Salkovskis et al., 2002)	Multiple hierarchical regression. Baron and Kenny (1086). Sobel test. Bootstrapping. (Preacher & Hayes, 2004); No	Negative affect fully mediated the relationship between ACEs and health anxiety. Direct effect of ACEs on health anxiety not significant after controlling for negative affect (*p* = 0.716). Bootstrapping estimates CI = (3,697 to −0. 9,186) and Sobel test, (z = 4.93, *p* < 0.001) confirmed statistical significance of IE. Trait anxiety fully mediated the relationship between ACEs and health anxiety. DE of ACEs on health anxiety not statistical significant (*p* = 0.51). Sobel test demonstrated statistical significance of IE (z = 4.45*, p* < 0.001).
Ross et al. (2019), USA	Community sample and students recruited through posters in the community and campus.	244; 75.10%; 20.80	Cross‐sectional	CTQ‐SF; Childhood maltreatment	Self‐compassion; Self‐compassion scale (Neff, 2003) Shame; Internalized Shame Scale (ISS; Cook, 1994, 2001)	None tested	Depression; Center for Epidemiological Studies Depression Scale‐Revised (CESD‐R; Eaton et al., 2004)	Multiple mediation model; Path models with R package covRobust (Wang & Raftery, 2002). Bootstrapping (95% CI); Yes	Significant IE of emotional abuse on depression through self‐compassion and shame (*b* = 0.084 [0.032], BC 95% CI [0.03–0.13), *p* = 0.008). DE of emotional abuse on shame (*β* = 0.131[0.049], 95% CI [0.04–0.23], *p* = 0.008). IE of emotional abuse on depression through shame (*b* = 0.089 [0.033], 95% CI [0.01–0.15]. = 0.008, *p* = 0.008) Significant effect of emotional neglect on depression through self‐compassion followed by shame (*b* = 0.178 [0.034], BC 95% CI [0.11–0.25] *p* < 0.001). Shame accounted for 14.8% and emotional abuse accounted for 1.5% of variance in depressive symptoms. Shame is a stronger predictor
Song et al. (2020), China	Undergraduate students recruited from two medical colleges in China.	7,643; 74.12%; 19.67	Cross‐sectional	CTQ‐SF; Childhood maltreatment	Coping styles; Coping style questionnaire (CSQ; Xiao & Dai, 2018).	None tested	Depression; Center for Epidemiological Studies Depression Scale (CES‐D; Radloff, 1977)	Multiple mediation model. Bootstrapping (95% CI), PROCESS (Hayes, 2013); Yes	IEs of child maltreatment on depression though problem solving (*b* = −0.913), self‐blame (*b* = 1.307), help‐seeking (*b* = − 0.493), problem avoidance (*b* = 0.104) and rationalization (*β* = 0.158) in the whole group. IEs of child maltreatment on depression though problem solving (*b* = −0.836), self‐blame (*b* = 1.261), help‐seeking (*b* = − 0.517), problem avoidance (*b* = 0.138) and rationalization (*b* = 0.173) in the female group. IEs of child maltreatment on depression through problem solving (*b* = −1.127), self‐blame *(b* = 1.468) and help‐seeking (*b* = −0.419) in male participants. Self‐blame had the strongest intervening effect (*b* = 1.307) in total. No intervening effect identified for fantasy.
Taylor et al. (2021), USA	College students recruited from a south‐western university.	504; 64.9%; 19.83	Cross‐sectional	ACE questionnaire; Childhood adversity	Repetitive negative thinking; Perseverative thinking questionnaire (PTQ; Ehring et al., 2011)	Executive control; Attentional control scale (ACS; Derryberry & Reed, 2002)	Anxiety/Worry; Penn state worry questionnaire (PSQW; Meyer et al., 1990)	Moderated mediation model. Bootstrapping (95% CI), PROCESS; No	Significant IE of ACES on anxiety symptoms through repetitive negative thinking (*b* = 1.15, BC 95% CI = [0.64–1.78]). Significant effect of attentional control‐focusing on the relationship between repetitive negative thinking and worry (*b* = 0.02, *p* < 0.05, BC 95% CI = [0.004–0.035]). Attentional control moderated the IE of ACEs on worry through repetitive negative thinking (*b* = 0.02, *p* < 0.05, BC 95% CI = [0.004–0.035]).
Wang et al. (2020), China	College students recruited from universities in China.	404; 53.2%; 20.12	Cross‐sectional	CTQ‐SF; Childhood trauma	Personality Traits‐ Neuroticism; The NEO‐Five Factor Inventory (NEO‐FFI; Costa and McCrae, 1992)	Resilience; Conner‐Davidson Resilience Scale (CD‐RISC; (Connor & Davidson, 2003)	Depression; Beck Depression Inventory–II (BDI‐II; Beck et al., 1996).	Multiple linear regression (Baron & Kenny, 1986). Bootstrapping (BC; 95% CI), PROCESS Macro (Hayes, 2013); Yes (gender, age)	Significant IE of childhood trauma on later depression through neuroticism (BC 95% CI [0.094–0.219]. Partial mediation (*b* = 0.271, *p* < 0.001). IE of low resilience on the relationship between childhood trauma and neuroticism (IE = 0.146, SE = 0.032, 95% CI = [0.091–0.216]). IE not significant when higher levels of resilience: (IE = 0.028, *SE* = 0.035, 95% CI = [−0.041–0.094]).
Watt et al. (2020), USA	Students recruited from a south‐east university in US.	404; 73.6%; 19.87	Cross‐sectional	ACE questionnaire; Childhood trauma (if 4 or >4 ACEs	Perceived social support; ENRICH Social Support Index (ESSI; Vaglio et al., 2004)	None tested	Depression; Patient Health Questionnaire (PHQ‐8; Kroenke et al., 2001) Anxiety; General Anxiety Disorder Scale (GAD‐7; Spitzer et al., 2006	SEM (using Mplus 7); Yes (age, gender, parents level of education, race or ethnicity)	IE of ACES (4 or >4) on depression (.789, *p* = 0.001) and anxiety (0.626, *p* = 0.002) through perceived social support.
Wells et al. (2014), USA	Students with no history of psychiatric disorders recruited from the University of Texas.	155; 53.5%; 18.8	Cross‐sectional	CTQ‐SF; Childhood abuse	Cognitive vulnerability/ information processing bias; Dysfunctional Attitudes Scale (DAS; Weissman & Beck, 1978) Scrambled Sentences Test (SST; Wenzlaff & Bates, 1998)	None tested	Depression; CES‐D (Radloff, 1977) (baseline depression scoring)	Hierarchical linear regression. PRODCLIN programme (MacKinnon et al., 2007) for Significance testing; No	Effect of childhood abuse on baseline depression non‐significant (*β* = 0.12, *t* = 1.48, *p* = 0.14). Significant mediational effect of child abuse on baseline depression scores through dysfunctional attitudes (95% CI = [0.014–0.0642]). Dysfunctional attitudes mediated the association of emotional maltreatment and depression (BC 95% CI = [0.002–0.087]). No significant effect was found for physical maltreatment Follow up mediation analysis showed that child abuse may contribute to cognitive vulnerability and this may lead to increased symptoms of depression (95% CI = [0.007–1.07]).
Wilson and Newins (2018), USA	Participants recruited through a mid‐Atlantic public university psychology subject pool.	336; 70.2%; 22.81	Cross‐sectional	CTQ Childhood maltreatment	Anxiety sensitivity; Anxiety Sensitivity Index‐3 (ASI‐3; Taylor et al., 2007)	None tested	PTSD Checklist for DSM‐5 (PCL‐5; Weathers et al., 2013)	Bootstrapping (BC; 95% CI), PROCESS Macro (Hayes, 2013); Yes (alcohol dependence, suicidal ideation)	Significant IE of child maltreatment on PTSD via anxiety sensitivity (*β* = 0.08, BC 95% CI [0.27–0.131]). DE of child maltreatment remained significant. Alcohol dependence, suicidal ideation entered in the model as covariates.

Abbreviations: ACE questionnaire, CDC‐Kaiser Permanente Adverse Childhood Experience Questionnaire; Felitti et al., 1998; BC, bias corrected; CI, confidence interval; CTQ, Childhood Trauma Questionnaire (Bernstein & Fink, 1998; Bernstein et al., 2003); CTQ‐SF, Childhood Trauma Questionnaire short form; DI, direct effect; IE, indirect effect; PTSD, post‐traumatic stress disorder.

### 2.4 Critical appraisal

Quality assessment was performed using a set of criteria which was adapted from a quality assessment tool previously utilized for critically evaluating mediation experimental studies (Cerin et al., 2009; Lubans et al., 2008; Mansell et al., 2013)] and subsequently modified to be utilized for observation studies (Lee et al., 2015). Additional criteria were added to the original tool to enable assessment of eligible research articles in terms of sampling procedures and bias, psychometric characteristics of independent variables, operationalization of mediators/moderators, reporting on the effect size, confidence intervals (CIs), and the variance accounted for in mediation models. The final assessment tool comprised 10 criteria (Table 6). Included studies were evaluated and given a score of 0 (weak), 1 (moderate) or 2 (strong) to each one of the criteria, while a total quality score was calculated for each study. A second rater critically appraised 30% of the included studies, with 83% agreement between raters.

### 2.5 Levels of scientific evidence

A Best Evidence Synthesis framework Rating System (BESRS) was utilized for the quality assessment of eligible studies. Following previous reviews using the same rating system (e.g., van Stralen et al., 2011), available information was synthesized taking into account three levels of scientific evidence: the quality of the study, the number of studies testing the same mediator and the outcome. According to this rating system, there is strong evidence when multiple studies of high quality have shown consistent findings, moderate evidence when one high quality and one or more lower quality studies display consistent findings and finally, insufficient evidence when one only study has provided evidence or when findings appear to be inconsistent in multiple studies.

## 3 RESULTS

### 3.1 Study selection

**FIGURE 1:**
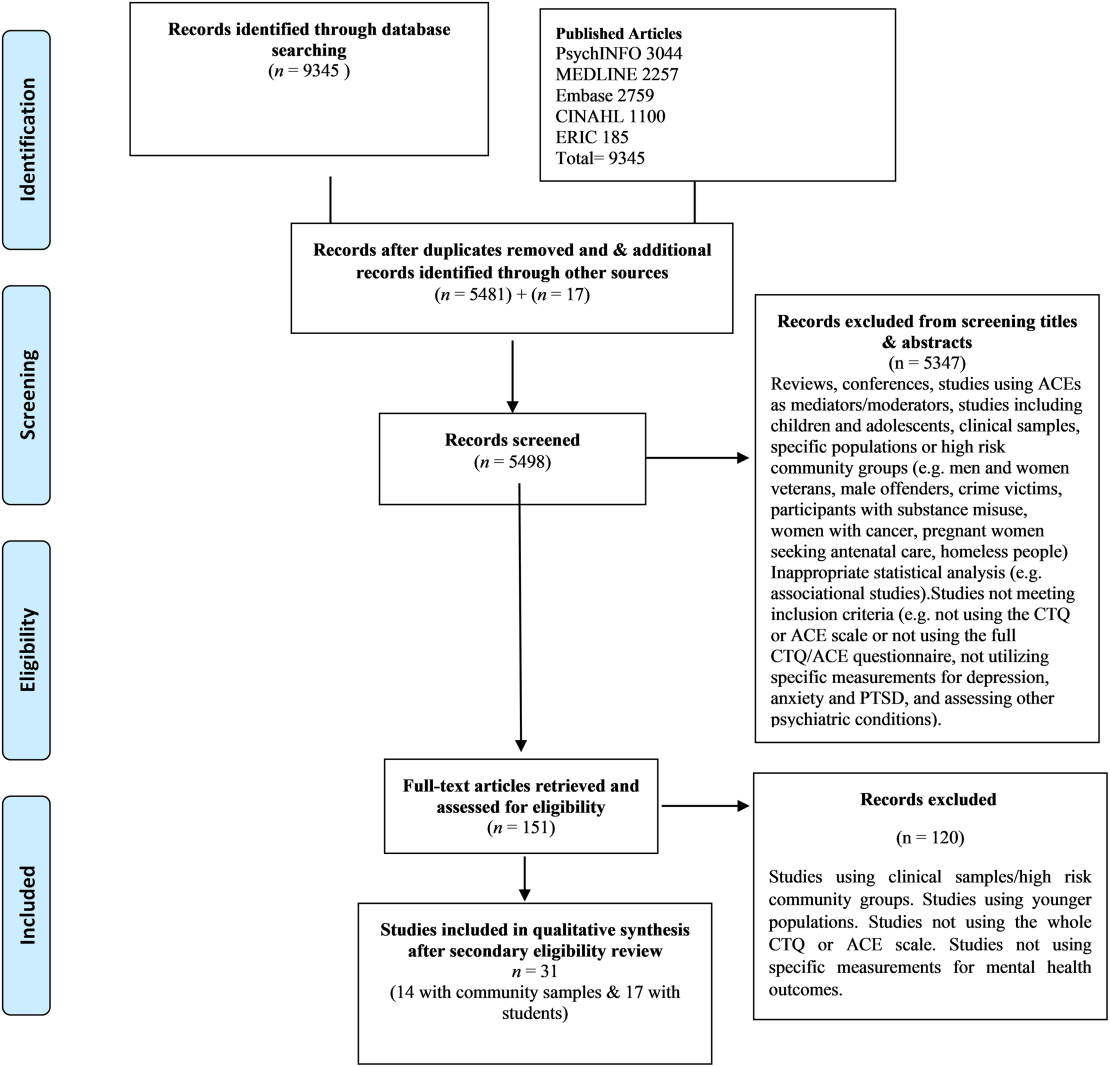
Study flow diagram showing process of study selection

The initial search strategy identified 9,345 research studies out of which 5,481 were retrieved following removal of duplicates. Screening of reference lists of the eligible articles and searching Google Scholar yielded additional 17 articles. In total, 5,498 records were screened out of which 151 full texts were examined. Finally, 31 complete texts meeting the predefined search criteria were retrieved for critical appraisal. Studies were also excluded if they used exclusively clinical samples or high‐risk community groups (e.g., veterans, male offenders, participants with substance misuse, etc.). See Figure 1 for study selection.

### 3.2 Study characteristics

All included studies were published between 2007 and 2020. Sample sizes ranged from *n* = 81 to *n* = 3,902 in the community‐based studies and *n* = 155 to *n* = 7,643 in the studies using student samples, representing a total sample size of *n* = 22,880. Total number of community participants (excluding students) was *n* = 9,415, while the number of participants in student samples was *n* = 13,465. Participants' mean age ranged from 18.60 to 74.90 years. The majority of the studies were carried out in the United States (*n* = 18, 58%), with papers also identified from Canada (*n* = 3, 10%), China (*n* = 3, 10%), Belgium (*n* = 1, 3%), Germany (*n* = 2, 6%), Brazil (*n* = 1, 3%), Indonesia (*n* = 1, 3%), Ireland (*n* = 1, 3%), Australia (*n* = 1, 3%) and South Korea (*n* = 1, 3%). Of the included studies, 68% (*n* = utilized the CTQ (Bernstein et al., 2003) and 32% (*n* = 10) utilized the ACE questionnaire (Felitti et al., 1998). Study characteristics and key findings are reported in Tables 1 and 2 (community and student samples, respectively).

### 3.3 Quality appraisal

The mean overall quality of the studies using community samples was moderate (63%), with a range from 45% to 90%. Mean overall quality for student studies was similar (57%), with a range from 30% to 80%. The majority of studies were rated moderate to strong.

All included studies provided adequate evidence for their rationale behind the specific mediated and moderated effects they explored in the relationship between ACEs and emotional well‐being in adulthood (first criterion). The majority of studies also provided adequate information on sampling procedures, inclusion/exclusion criteria and setting (second criterion). The reviewed studies used either paper–pencil surveys or online survey platforms for data collection, such as Amazon Mechanical Turk or Qualtrics (e.g., Hayward et al., 2020; Watt et al., 2020). Some biases were acknowledged within studies, such as sampling based on specific demographic characteristics (e.g., age and gender), indicating results may not be generalizable to general populations (e.g., Jardin et al., 2019; Stevens et al., 2013). Furthermore, the use of homogenous student samples could limit the generalizability of results as a function of skew towards younger ages (e.g., Brown et al., 2016).

The majority of studies utilized psychometrically sound validated measures, although studies varied in the extent to which they reported validity and internal reliability. In some cases, information about psychometric properties was provided by computing and reporting internal reliability within the study (e.g., McQuaid et al., 2015; Stevens et al., 2013) or/and by providing references (e.g., Evans, Steel, & DiLillo, 2013). There was some variability around the reporting of the psychometric properties of translated measures. The measures used to assess mental health outcomes varied across studies. A total of 23 self‐report questionnaires were utilized for the measurement of outcomes, with the Beck Depression Inventory (BDI; Beck et al., 1996) most frequently used (*n* = 8).

A minority of studies (Kogan et al., 2021; Ross et al., 2019; van Assche et al., 2020; Wong et al., 2019) reported an a priori power analysis before data collection (fourth criterion). Proposed mediating variables were adequately defined by researchers in the majority of the studies, and valid measurements were utilized in most cases. Therefore, the majority of the studies received strong ratings in this (fifth) criterion. Methods of data analysis employed were evaluated based on their power and statistical significance (sixth criterion). Therefore, the causal steps approach was rated weak given its low power (Fritz & MacKinnon, 2007); studies using the Sobel test (Sobel, 1982) in conjunction with the Baron and Kenny approach were evaluated as moderate, and finally, studies used estimation procedures for direct and indirect effects to test hypotheses regarding mediating effects (e.g., SEM and bootstrapping) (Agler & de Boeck, 2017; Hayes, 2009) were rated strong on this criterion. Of the total studies, 28 used SEM or process analyses with bootstrapping. The majority of the studies reported effect sizes adequately and included CIs (seventh criterion).

Mediation models assume that mediators are affected by the exposure variable and that changes in mediators are associated with changes in the outcome. Since the majority of studies were cross‐sectional and data were gathered at a single time point, the temporal ordering of variables was speculative (eighth criterion).The majority of papers argued that longitudinal research would be required with a view to exploring how associations between variables play out with time. Approximately half of the included community‐based and one third of the student‐based studies reported the variance accounted for in their mediation models (Fairchild et al., 2009) (ninth criterion). Finally, the issue of potential confounding variables or covariates was considered in 6 out of 14 community‐based studies and in 8 out of the 17 studies featuring students.

### 3.4 Synthesis of psychosocial mechanisms

The majority of included studies (*n* = 25; 81%) explored factors that mediated or moderated the relationship between child adversity and depression; 12 studies (39%) investigated intervening variables in the relationship between child adversity and anxiety, and six studies (19%) examined mediators in the relationship between adversity in childhood and trauma. In some studies, the role of biological intervening variables was also tested (e.g., Kogan et al., 2021). These variables were outside the scope of the current review. The majority of the studies reported significant mediation effects. Key results of analyses per category of mental health outcomes are summarized below.

#### 3.4.1 Mediators/moderators of the relationship between childhood adversity and depressive symptoms

Twenty‐four studies investigated the role of potential psychological mediators, and two tested social/interpersonal mediators. Of the studies that reported on putative psychosocial mediators in the relationship between child adversity and depression, 22 found that there was significant mediation, indirect effect and partial or complete mediation (Table 3).

**TABLE 3:** Summary of key research findings for depression

Study (author, year)	Mediation/moderation results
Fitzgerald and Gallus (2020)	Significant indirect effect of child maltreatment on depression via emotional support from family and romantic partners but not friends.
Hayward et al. (2020)	Significant indirect effect of child adversity through self‐concept clarity on depression. Both self‐concept clarity and intolerance of uncertainty mediated the link between child adversity and mental health outcomes.
van Assche et al. (2020)	Significant indirect effect of emotional neglect on depression through attachment anxiety. No direct effect found between emotional neglect and depression.
Kogan et al. (2021)	ACEs predicted young adult contextual stress which in turn forecast increases in defensive/hostile schemas. Defensive/hostile schemas predicted increases in social developmental risk factors which were a proximal antecedent of depressive symptomatology and substance abuse no direct effect of ACEs on relational schemas reported. ACEs affect mental health outcomes indirectly through contextual contemporary factors.
Nowalis et al. (2020)	Anxious attachment to primary caregiver significantly moderated the child maltreatment–depression relationship. No significant effect identified for avoidant attachment to primary caregiver and for anxious or avoidant attachment to secondary caregiver.
Song et al. (2020)	Indirect effect of child maltreatment on depression though problem solving, self‐blame, help‐seeking, problem avoidance and rationalization.
Wang et al. (2020)	Significant indirect effect of childhood trauma on later depression through neuroticism. Indirect effect of low resilience on the relationship between childhood trauma and neuroticism.
Watt et al. (2020)	Indirect effect of ACES (4 or >4) on depression through perceived social support.
Berman et al. (2019)	Significant indirect effect of child maltreatment (abuse and neglect subscales) and household dysfunction on depressive symptomology via negative core beliefs
Cantave et al. (2019)	Significant indirect effect of maltreatment on depression via emotion‐oriented coping strategies. No indirect effect found for task‐oriented and avoidance‐oriented coping strategies. Avoidance‐oriented and emotion‐oriented coping strategies did not moderate the maltreatment–depression relationship. Task‐oriented coping strategies moderated the maltreatment‐depressive symptoms association.
Gomes Jardim et al. (2019)	Significant indirect effect of childhood maltreatment on depression via neuroticism and extraversion. Total mediation effect. Non‐significant effect of openness. Significant indirect effect of early adversity on depression via agreeableness and conscientiousness. Partial mediation effect.
Klumparendt et al. (2019)	Significant total indirect effect of childhood maltreatment on depression through mediators & not significant direct effect of childhood maltreatment on depression in total model. Total mediation. Emotion regulation, depressogenic attributional style and post‐traumatic symptom severity mediated the relationship between childhood maltreatment and depression. Emotion regulation: Strongest indirect effect of limited access to emotion regulation strategies and lack of emotional clarity. Significant indirect effect through post‐traumatic symptoms. Significant indirect effect through depressogenic attribution style. No indirect for attachment anxiety or avoidance
Makriyianis et al. (2019)	Significant indirect effect of ACEs on depression via inflexibility.
Mishra and Marceau (2020)	The effect of sexual abuse on depression partially explained by perceived stress levels in middle life. The effect of physical and emotional maltreatment on depression fully mediated by both perceived stress and cortisol.
Wong et al. (2019)	Significant indirect effect of ACEs on depression through self‐concept clarity and perceived stress.
Ross et al. (2019)	Significant indirect effect of emotional abuse on depression through self‐compassion and shame.
Corcoran and McNulty (2018)	Significant indirect effect of childhood adversity on depression through attachment anxiety general, attachment anxiety friend and attachment avoidance mother.
Gong and Chan (2018)	Significant indirect effect of physical abuse, emotional abuse, and sexual abuse on depression through early maladaptive schemas.
Kaloeti et al. (2019)	Resilience did not mediate the ACEs–depression relationship.
Kim et al. (2017)	Significant direct and indirect effect of childhood maltreatment on mood through rumination.
McCormick et al. (2017)	Significant indirect effect of ACEs on depressive symptoms through the meaning struggles.
Crow et al. (2014);	Significant indirect effect of childhood emotional abuse on depression through emotion dysregulation. Emotional abuse stronger predictor of adult depression.
McQuaid et al. (2015)	Iindirect effect of childhood trauma on depression through perceived discrimination. Partial mediation. Significant direct effect of childhood trauma on depression. Moderated mediation analyses showed that the intervening role of discrimination was stronger when levels of outgroup unsupport were higher. Ingroup unspport did not moderate the mediated relationship. Multiple mediation analyses: Emotion focused coping mediated the relationship between childhood trauma and depression. The path between emotion‐focused coping and depression was moderated by both ingroup and outgroup unsupport.
Wells et al. (2014)	Significant mediational effect of child abuse on baseline depression scores through dysfunctional attitudes. Dysfunctional attitudes mediated the association of emotional maltreatment and depression. No significant effect was found for physical maltreatment

##### Psychological mechanisms

Three studies examined the role of coping strategies as putative mediating variables of the childhood adversity–depression relationship. All identified emotion‐oriented coping strategies as mediating the early adversity–depression relationship (Cantave et al., 2019; McQuaid et al., 2015; Song et al., 2020). Task‐oriented coping strategies were found to function as a moderator in one study (Cantave et al., 2019). Song et al. (2020) tested the role of six coping strategies, via multiple mediation, reporting that problem solving, help‐seeking behaviour, self‐blame, problem avoidance and rationalization mediated the relationship between child maltreatment and depression. Of note, problem avoidance and rationalization did not mediate the effect among male participants. Self‐blame was identified as the mechanism with the strongest mediating effect.

In a cross‐sectional study (Crow et al., 2014), the authors hypothesized that emotion dysregulation mediates the relationship between childhood adversity and adult depression. Similarly to other studies (e.g., Klumparendt et al., 2019), study results lent support for its intervening effect on the relationship between emotional abuse and adult depression. Relatedly, Brown et al. (2016) investigated the role of alexithymia (an emotion regulation deficit) and found a significant indirect effect of emotional neglect on depression, indicating that alexithymia may be a potential mechanism linking early adversity (emotional neglect category) and depression.

Two studies (Hayward et al., 2020; Wong et al., 2019) reported that self‐concept clarity may be a potential mediator linking early adversity and adult depression. Notably, when Wong et al. (2019) added self‐esteem in the model, both the indirect effects through self‐concept clarity and self‐esteem were significant for depression, with the effect of self‐esteem being larger. Additionally, Hayward et al. (2020) suggested that intolerance of uncertainty may also mediate the early adversity–depression relationship.

Four studies suggested a mediating effect of specific depressogenic cognitive styles (Berman et al., 2019; Kim et al., 2017; Makriyianis et al., 2019; Wells et al., 2014). Specifically, a study by Kim et al. (2017) provided support for the mediating role of rumination and identified gender differences, with the observed effect predominating in female participants. Similarly, Berman et al. (2019) reported that negative beliefs mediated the child maltreatment/household dysfunction and internalizing symptoms relationship. Makriyianis et al. (2019) provided support for the mediating role of psychological inflexibility, while Wells et al. (2014) reported a significant mediational effect of dysfunctional cognitions in the emotional abuse–depression relationship.

Three studies focused on the role of attachment as a potential intervening variable in the relationship between early adversity and adult depression (Corcoran & McNulty, 2018; Nowalis et al., 2020; van Assche et al., 2020), reporting significant mediating and moderating effects for attachment anxiety. No significant mediation/moderation effects were identified for attachment avoidance (Nowalis et al., 2020; van Assche et al., 2020). However, Corcoran and McNulty (2018) suggested an indirect effect, reporting that the avoidant attachment style with one's mother mediated the relationship between child adversity and depression.

The role of early maladaptive and relational schemas was explored in two studies Gong & Chan, 2018; Kogan et al., 2021). Kogan et al. (2021) tested a developmental model exploring the role of relational schemas and found that they were not directly associated with ACEs. The study showed that contextual stressors predicted these schemas, which in turn predicted social developmental factors which constituted antecedents of symptoms of depression and substance abuse. Contrastingly, the study by Gong and Chan (2018) yielded indirect effects of all types of maltreatment on psychological distress via early maladaptive schemas.

Klumparendt et al. (2019) used a more integrated approach and applied a mediation analysis with a view to investigating the role of attachment, emotional regulation, attributional style and PTSD, which were identified as important mediators. Unlike the studies by Corcoran and McNulty (2018), Nowalis et al. (2020) and van Assche et al. (2020), this study reported no significant findings for attachment anxiety. However, similarly with the aforementioned studies, Klumparendt et al. (2019) reported no significant findings for attachment avoidance.

Regarding the role of personality, two studies yielded similar results, indicating that neuroticism may be operating as a mediating variable in the link between ACEs and depression (Gomes Jardim et al., 2019; Wang et al., 2020). Additionally, the study by Gomes Jardim et al. (2019) reported that neuroticism and extraversion had a total mediation effect while agreeableness and conscientiousness had a partial effect. No effect was found for openness. The study by Wang et al. (2020) demonstrated that resilience moderated the association between adversity and depression. Contrastingly, Kaloeti et al. (2019) performed a path analysis to investigate the function of resilience and did not report a significant indirect effect.

Ross et al. (2019)) revealed a significant indirect effect of emotional abuse on depression through self‐compassion and shame. Using a multiple mediation model, emotional abuse was strongly associated with lower self‐compassion scores which predicted shame, which then predicted depressive symptoms. They also reported a significant indirect effect of emotional neglect on depression through self‐compassion and shame. Finally, McCormick et al. (2017) examined the role of religious and spiritual struggles. ‘Meaning struggles’ were identified as a mechanism through which ACEs had an indirect effect on depression. Mishra and Marceau (2020) explored the mechanistic effects of a psychological (perceived stress) and a biological factor (dysregulated stress system) and reported an indirect effect of maltreatment exposure on midlife depression via perceived stress (and cortisol).

##### Social/interpersonal mechanisms

Three studies investigated social/interpersonal mechanisms mediating the early adversity and depression link. Using SEM analysis to test mediating effects of emotional support, Fitzgerald and Gallus (2020) identified support from family and romantic partners as mediators of the child maltreatment–depressive symptoms relationship. Consistent with previous research findings (Stafford et al., 2011), the results suggested that emotional support from friends did not have an intervening effect. Finally, Watt et al. (2020) investigated the effect of health behaviours and perceived social support in the relationship between adversity in childhood and depression in college students, concluding that perceived social support partially mediated the above relationship.

#### 3.4.2 Mediators/moderators in the relationship between childhood adversity and anxiety symptoms

Of the total studies, 12 (five community‐based and seven with student‐based) investigated the role of potential intervening variables in the child adversity–anxiety relationship. All studies reported on several psychosocial mediators of the relationship between child adversity and anxiety, revealing that there was an indirect effect, significant mediation or partial mediation (Table 4). Anxiety symptoms were measured with a variety of outcome measures, among which the General Anxiety Disorder Scale (GAD‐7) was the most commonly used (33% of studies).

**TABLE 4:** Summary of key research findings for anxiety

Study (author, year)	Mediation/moderation results
Fitzgerald and Gallus (2020)	Significant indirect effect of child maltreatment on social anxiety through emotional support from friends and romantic partners.
Hayward et al. (2020)	Significant indirect effect of child adversity through self‐concept clarity and intolerance of uncertainty on generalized anxiety, social anxiety as well as obsessive compulsive disorder symptoms. Both self‐concept clarity and intolerance of uncertainty mediated the link between child adversity and mental health outcomes.
van Assche et al. (2020)	Significant indirect effect of emotional neglect on late life anxiety through attachment anxiety. No direct effect found between emotional neglect and anxiety.
Watt et al. (2020)	Indirect effect of ACES (4 or >4) on anxiety through perceived social support.
Berman et al. (2019)	Significant indirect effect of child maltreatment (abuse and neglect subscales) and household dysfunction on anxiety via negative core beliefs
Makriyianis et al. (2019)	Significant indirect effect of ACEs on anxiety via inflexibility.
Taylor et al. (2021)	Significant indirect effect of ACES on anxiety symptoms through repetitive negative thinking. Attentional control moderated the indirect effect of ACEs on worry through repetitive negative thinking.
Corcoran & McNulty, 2018	Significant indirect effect of childhood adversity on anxiety via attachment anxiety‐general.
Gong and Chan (2018)	Significant indirect effects of physical abuse, emotional abuse, and sexual abuse on anxiety through early maladaptive schemas.
Reiser et al., 2014	Negative affect fully mediated the relationship between ACEs and health anxiety. Trait anxiety fully mediated the relationship between ACEs and health anxiety.

##### Psychological mechanisms

Self‐concept clarity and intolerance of uncertainty were investigated as potential mediators on the path between ACEs and social anxiety, generalized anxiety and obsessive compulsive symptoms (Hayward et al., 2020). Hayward et al. (2020) reported that both self‐concept clarity and intolerance of uncertainty had a statistically significant indirect effect. Similarly, Wong et al. (2019) demonstrated a significant indirect effect of ACEs on perceived stress via self‐concept clarity and self‐esteem.

Two studies investigated the role of repetitive negative thinking or rumination (Kim et al., 2017; Taylor et al., 2021). Research findings revealed that repetitive negative thinking mediated the early adversity–adult anxiety relationship. Kim et al. (2017) found that the effect was stronger among female participants. Taylor et al. (2021) also demonstrated that heightened attentional control moderated the indirect effect of negative repetitive thinking on the ACEs–anxiety relationship, indicating that it may be a risk factor for anxiety. Furthermore, two studies demonstrated that early maladaptive schemas and negative core beliefs mediated the association between maltreatment in childhood and adult anxiety (Berman et al., 2019; Gong & Chan, 2018).

The role of attachment was examined by van Assche et al. (2020), reporting a significant indirect effect of early adversity on anxiety later in life via attachment anxiety. No significant findings were found for attachment avoidance.

Several studies tested the role of affect in the context of early negative experiences. Brown et al. (2016) tested the role of alexithymia using a student sample and found a significant indirect effect, indicating that alexithymia may be a potential mechanism linking early adversity and anxiety. Reiser et al. (2014) demonstrated that negative affect and trait anxiety served as full mediators on the ACEs–anxiety relationship. Finally, Makriyianis et al. (2019) investigated the potentially intermediary function of psychological flexibility/inflexibility and revealed that psychological inflexibility mediated the ACEs–anxiety link.

##### Social/interpersonal mechanisms

Fitzgerald and Gallus (2020) explored the role of emotional support and reported a significant indirect effect of childhood maltreatment on social anxiety via emotional support from romantic partners and friends. No effect was identified for support from family. Similarly, Watt et al. (2020) found that perceived social support partially mediated the childhood adversity–anxiety relationship.

#### 3.4.3 Mediators/moderators of the relationship between childhood adversity and trauma symptoms

Of the total number of included studies, six investigated the role of potential intervening variables in the association between child adversity and trauma symptoms. They reported on five mediators and one moderator in the relationship between child adversity and trauma symptoms, all yielding significant effects (Table 5).

**TABLE 5:** Summary of key research findings for trauma symptoms

Study (author, year)	Mediation/moderation results
Berman et al. (2019)	Significant indirect effect of child maltreatment (abuse and neglect subscales) on post‐traumatic symptoms via negative core beliefs.
Wilson and Newins (2018)	Significant indirect effect of child maltreatment on post‐traumatic symptoms via anxiety sensitivity.
McCormick et al. (2017)	Significant indirect effect of ACEs on post‐traumatic symptoms through the meaning struggles subscale of the Religious and Spiritual Struggles Scale (RSSS).
Evans, Steel, and DiLillo (2013)	Perceived social support (PSS) from family predicted moderation of the relationship between physical abuse, emotional abuse, emotional neglect and trauma. PSS from family did not moderate the child maltreatment–trauma symptoms for men. PSS from friends did not moderate the child maltreatment–trauma link for both men and women.
Stevens et al. (2013)	Significant indirect effect of childhood trauma on post‐traumatic symptoms through emotion regulation. Significant indirect effect of child abuse on post‐traumatic symptoms though interpersonal violence and social support. Emotion regulation mediates the relationship between child abuse and trauma. Effect significant after adjusting for social support and interpersonal violence. Social support & interpersonal violence mediates the relationship between child abuse and trauma. The effect of emotion regulation was stronger.
Browne and Winkelman (2007)	Indirect effect of childhood trauma on trauma symptoms via cognitive distortions. Associations found between childhood trauma and attachment dimensions which on their own were not significantly associated with trauma symptoms.

**TABLE 6:** Critical appraisal assessment tool

Study	Did the study cite a theoretical framework?	Was sufficient information provided about sampling procedures, inclusion/exclusion criteria and setting? Was there a selection bias?	Were psychometric characteristics of tools used to measure exposure, mediator and outcome variables reported?	Did the study report a power calculation? If so, was the study adequately powered to detect mediation?	Were mediators/moderators operationalized?	Were statistically appropriate/ acceptable methods of data analysis used and sufficient information provided?	Was effect size adequately reported including confidence intervals (CI)?	Did the study ascertain whether changes in the exposure variable preceded changes in the mediator variable and whether changes in the mediator variable (s) preceded changes in the outcome variables?	Was the proportion of the total effect mediated by the intervening variable reported?	Did the study control for possible confounding factors or covariates?	Total score (%)

##### Psychological mechanisms

Stevens et al. (2013) tested the possible mediating effects of emotion regulation difficulties and demonstrated a significant indirect effect, indicating that emotion regulation might be a significant mechanism in the pathway between early adversity and post‐traumatic stress symptoms in adulthood. Two studies investigated the possible intervening effect role of cognitive distortions and negative core beliefs (Berman et al., 2019; Browne & Winkelman, 2007) and revealed a strong path from early adversity to traumatic symptoms in adulthood through the suggested mediators. Browne and Winkelman (2007) examined the role of attachment which was not found to mediate the early adversity–trauma symptoms relationship. McCormick et al. (2017) carried out analysis of data collected from 512 undergraduate students reporting that meaning making had an intervening effect on the child adversity–PTSD relationship.

Finally, a study by Wilson and Newins (2018) examined anxiety sensitivity as a putative mediator in the child maltreatment–trauma symptoms relationship while entering in the model suicidal ideation and alcohol dependence as covariates. The study reported an indirect effect of child maltreatment on PTSD symptoms via anxiety sensitivity.

##### Social/interpersonal mechanisms

Evans, Steel, and DiLillo (2013) examined the role of perceived social support reporting that, perceived social support from family moderated the association between moderate severity physical abuse, emotional abuse and emotional neglect and trauma symptoms. However, this held only for women and support from friends did not emerge as a moderator. These findings echo Fitzgerald and Gallus (2020), who did not find evidence of emotional support from friends mediating the relationship between childhood adversity and depression. Stevens et al. (2013) tested the role of social support and interpersonal violence, demonstrating that both variables significantly mediated the effects of childhood abuse on PTSD later in life.

## 4 DISCUSSION

### 4.1 Summary of findings

The current review synthesized evidence regarding putative mediators and moderators of the relationship between robustly validated measures of child adversity (ACE scale or the CTQ) and mental health. Consistent with previous findings, the current review supports existing evidence for the link between early adversity and various forms of common psychopathology (e.g., Green et al., 2010; Hughes et al., 2017; Riedl et al., 2020) indicating that child adversity constitutes a transdiagnostic risk factor in the development and maintenance of poor mental health in adulthood. Additionally, there is evidence from the reviewed studies that there are multiple variables mediating and moderating this relationship, to varying levels of strength. Overall, the present review revealed significant intervening effects, suggesting specific pathways linking adversity with psychopathology, but the majority of mediators were investigated in single studies only. Notably, the majority of the studies focused on depression and anxiety and less on trauma symptoms.

With regard to the identified levels of scientific evidence, research findings found strong evidence for the role of both emotion‐oriented coping strategies and attachment anxiety upon the association between adversity in childhood and depression later in life. Furthermore, three high‐quality studies explored the role of attachment avoidance, finding no evidence for mediation or/and moderation. Therefore, there is strong evidence for the lack of effect of attachment avoidance upon the child adversity–mental health association. Moderate evidence was also found for the intervening effect of emotion regulation, perceived social support (general, family, partner and friends), self‐concept clarity and neuroticism in the relationship between adversity in childhood and depression. Neither strong nor moderate evidence for any of the examined variables was identified in studies that tested mediation and moderation models in the relationship between child adversity and trauma symptoms or anxiety. Overall, there is insufficient evidence for the majority of the putative mediating and moderating psychosocial variables as they were analysed in single studies.

**FIGURE 2:**
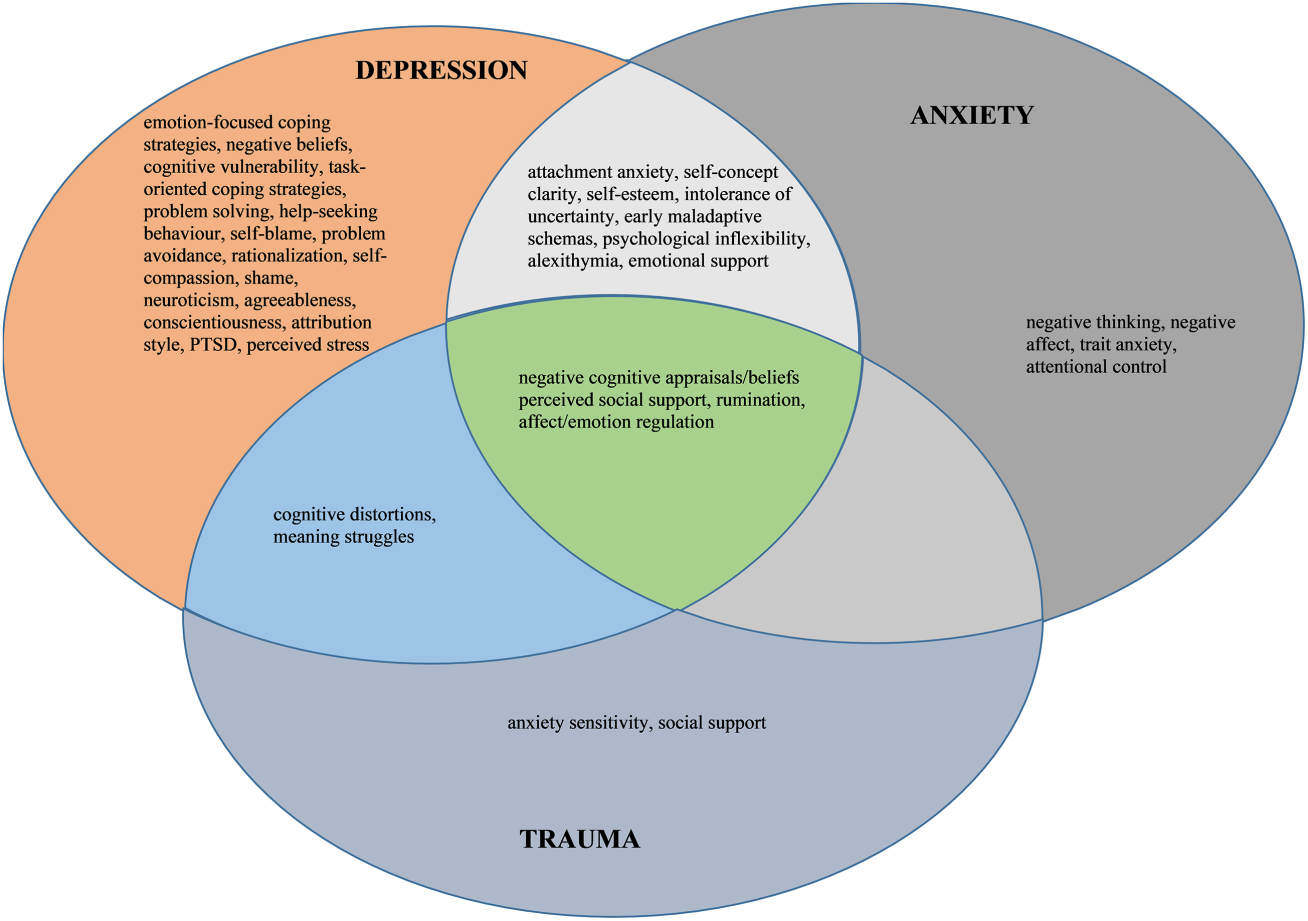
Mechanisms linking childhood adversity and psychopathology

On the basis of evidence from the papers included in the current review, we identify a number of candidate transdiagnostic mechanisms, particularly social support, cognitive appraisals and emotion regulation (Figure 2). These mechanisms can be conceptualized as either risk or protective factors, operating across a spectrum of mental health difficulties, without disorder specificity for disparate disorders (Cludius et al., 2020; Compas et al., 2014; Conway et al., 2016). Other mechanisms explored in the reviewed studies were found to be relevant to anxiety and depression only (e.g., attachment anxiety and psychological inflexibility), while others were shown to be relevant to specific disorders (e.g., self‐compassion and shame for depression and attentional control for anxiety). Of note, some seemingly disorder‐specific mechanisms may be in fact transdiagnostic but are as yet untested (e.g., shame) or tested against a different measurement of adversity. From a lifespan perspective, the current findings are consistent with similar research in early adversity and psychopathology in adolescence, where the mediating role of processes identified in the current review, including emotion regulation, social support, cognitive appraisals and self‐concept, has also been identified (Dhondt et al., 2019; John et al., 2017; Lansing et al., 2019; Negriff et al., 2019; Paredes & Calvete, 2014).

### 4.2 Strengths and limitations

We acknowledge several limitations in the identified papers. The use of a large number of homogenous samples may limit the external validity of study findings, as could the use of non‐random sampling (Banerjee & Chaudhury, 2010). The majority of the included studies also used volunteer selection introducing potential sampling bias or non‐response bias (Cheung et al., 2017). Several studies used online recruitment platforms, such as Qualtrics and Amazon's Mechanical Turk, as they offer advantages over convenience sampling, such as student subject pools, by providing more diverse study participants. Nevertheless, there is evidence that even this form of recruitment remains limited in terms of sample diversity (Chandler et al., 2019; Walters et al., 2018). Considering and adjusting for mediator–outcome confounding variables also increases the likelihood of validity of inferences regarding both direct and indirect effects or about a mediation relationship (VanderWeele, 2016). However, only a small number of studies controlled for confounders and discussed the extent to which controlling for potential confounders may have affected the power of the study.

Further, in all studies, early negative experiences were measured retrospectively. It has been argued that this way of measuring past adversity may introduce potential measurement error into the findings, in the direction of either under‐ or over‐reporting the impact of early adverse experiences on individual well‐being (Colman et al., 2016; Hardt & Rutter, 2004). Of note, studies that compared retrospective and prospective methods of measuring ACEs varied between moderate (Reuben et al., 2016; Tajima et al., 2004) and low agreement (Baldwin et al., 2019). These findings should be interpreted in context and rather than indicating inadequate validity of retrospective measures, may instead reflect complementarity of both retrospective and prospective methods of measuring adversity (Newbury et al., 2018; Reuben et al., 2016). In addition, the use of cross‐sectional designs in most studies means temporal precedence (a requirement of mediation modelling) cannot be accurately determined (Pirlott & MacKinnon, 2016). However, despite these limitations, well‐designed cross‐sectional mediation studies can still give insight into pathways linking early adversity and adult mental health (Fairchild & McDaniel, 2017).

The systematic review also has several limitations. First, including only studies that utilized two well‐validated measures of childhood adversity reduced variance, gave a clear, operationalization of adversity, and imposed a degree of homogeneity on a heterogeneous literature. However, equally, this was achieved at the cost of excluding studies measuring adversity using other measures of adversity exposure. Second, including only generalized non‐clinical population was intended to increase specificity by reducing confounding variables emerging from specific or high‐risk populations, but at the cost of impacting on generalizability to these same specific populations. Third, the current review included only published peer reviewed studies. Fourth, we did not include research on biomarker mediators of child adversity and mental health, although there is some evidence of their role in affective disorders link (e.g., Koss & Gunnar, 2018). Finally, although we constrained the operationalization of childhood adversity, we did not do the same for outcomes. Consequently, there is likely measurement variance due different operationalizations of mental health outcomes.

### 4.3 Implication for research, practice and theory development

From a research perspective, it is important that future research on moderating and mediating factors relating to ACEs employ longitudinal and experimental designs, larger samples and robust statistical modelling. Importantly, the role of these factors may be dependent on the type of the adversity, suggesting a focus upon disentangling the unique contribution of different types of adversity and different mechanisms upon transdiagnostic mental health outcomes. For instance, some of the reviewed studies provided evidence for the role of child emotional abuse (e.g., Crow et al., 2014; Evans, Li, & Whipple, 2013) in the development of depressive, anxiety and trauma symptoms, while others highlighted the role of emotional neglect (e.g., Brown et al., 2016; van Assche et al., 2020). Additionally, it is possible that some of the mechanisms explored in the reviewed studies may also explain the link between negative experiences in adulthood and psychopathology. Therefore, future research could focus on exploring the role of possible psychosocial mechanisms in relation to adversity experienced across the life span, rather adversity in one given developmental period (e.g., childhood). Furthermore, the questionnaires utilized in the included studies predominantly focus on relational types of adverse experiences that themselves largely occur within the familial environment. However, there are diverse types of adverse experiences that could also be the focus of future research, such as violence in the community, bullying at school, racial discrimination, medical crisis and war (Cross et al., 2015; Halevi et al., 2017; Strasshofer et al., 2018).

The current review focused on anxiety, depression and PTSD/trauma on the basis of the potential for common underlying transdiagnostic processes (Gardner et al., 2019; Teicher & Samson, 2013). Given the number and breadth of studies on ACEs and common mental health outcomes, meta‐analytic studies of potential mediating variables in the relationship between child adversity and mental health using both clinical and community samples could be useful. However, there are methodological challenges herein due to the heterogeneity of potential psychosocial mechanisms, outcome measures, mediation analysis approaches and hence, methods of reporting results, merit consideration.

With regard to clinical implications, we highlight the need for early identification and implementation of primary, secondary and tertiary interventions with a view to targeting adversity at various levels (Thoresen & Olff, 2016). There is evidence for key mediating processes, such as social support, emotion regulation, and cognitive processes, coping strategies, self‐esteem and attachment, that could be taken into account in screening procedures, assessment, and design of clinical or preventative interventions. Therefore facilitating improvement in these psychological targets could produce corresponding improvement in mental health symptoms. Specifically, these could be targeted and used as an adjunct to traditional approaches to treating depression, anxiety and PTSD in populations with experiences of adversity in childhood (e.g., cognitive‐behavioural therapy [CBT]; Cuijpers et al., 2016) or form the basis of stand‐alone interventions.

On the basis of the current findings, targeting social inclusion and support via group‐based interventions could also impact on affective symptoms in people with a history of adverse experiences in childhood. Furthermore, since emotion dysregulation appears to be one of the mechanisms that could explain the child adversity–psychopathology association (Crow et al., 2014; Klumparendt et al., 2019; Stevens et al., 2013), therapists working with sufferers of abuse in the past should remain mindful to possible emotion regulation deficits and facilitate the development of emotion regulation skills, if needed, during treatment. There is preliminary evidence that participation in programmes designed to facilitate development of emotion regulation skills in people with experiences of adversity can promote people's resilience and psychological well‐being (Cameron et al., 2018). Finally, digital or peer‐led interventions and/ or preventive programmes in the community could encourage a greater use of task‐oriented strategies to help tackle the maladaptive use of emotion‐oriented coping strategies shown to explain the child adversity–affective disorders link (Cantave et al., 2019; McQuaid et al., 2015).

The use of non‐clinical community‐based samples in the review improves generalizability to the community and, as such, can help inform public mental health approaches and the design of preventative programmes. From a preventative perspective, our findings highlight the role of coping as a mediator, suggesting that a more general focus on improving children's self‐esteem, self‐concept clarity, task‐focused coping strategies and teaching adaptive ways to express and regulate their emotions could be targeted as part of early intervention and prevention programmes designed to be delivered by mental health professionals in various settings, including schools. Furthermore, children and adolescents who have suffered adverse experiences within their familial environment often do not seek support early, leading to help‐seeking difficulties and a long duration of under‐treated symptoms, further supporting the impetus for early intervention and the development of preventative programmes.

### 4.4 Conclusion

Overall, our review highlights the need to prevent ACEs and develop interventions aiming to provide support to individuals exposed to early adversity. The wide range of potential mediators examined in the included studies foregrounds the difficulties in unifying these factors within a single theoretical framework. Indeed, the association between child adversity and psychopathology is multifactorial and context‐related. We emphasize the importance of further research on the field with a view to garnering more evidence‐based information about factors that influence the link between childhood adversity and specific mental health outcomes. Finally, another crucial issue is the lack of consensus criteria upon the way adversity and trauma are conceptualized. These criteria should be established and agreed upon with a view to supporting intervention, research and policy development.

## CONFLICT OF INTEREST

The authors declare that there is no conflict of interest.

## Supporting information




**Appendix S1.** Supporting Information
