# Supplementary material for: Deconstructing pathways to resilience: A systematic review of associations between psychosocial mechanisms and transdiagnostic adult mental health outcomes in the context of adverse childhood experiences
Source: Clin Psychol Psychother. 2022 Mar 18;29(5):1626–54. doi: 10.1002/cpp.2732 (PMC9790303; doi:10.1002/cpp.2732)
Supplement: Supplementary file 1 — Appendix S1. Supporting Information [file CPP-29-1626-s001.docx]

Systematic Review: Search Strategy

--------------------------------------------------------------------------------

1     ("adverse child* experience*" or "child* trauma" or "child* traum* experience*" or "negative child* experience*" or "child* maltreatment" or "child* abuse" or "early life experience*" or "child* advers*" or "early advers*").mp. (136904)

2     (mediat* or moderat* or "structural equation model*" or SEM or "Baron and Kenny" or "product* of coefficient*" or "difference* in coefficient*" or "process of change" or sobel or "causal pathway*" or "indirect effect*" or "process variable*" or "mechanism* of change" or bootstrap* or "psychological mechanism*").mp. (5163362)

3     (depress* or anxi* or phobia* or PTSD or "post-traumatic stress" or "post-traumatic symptom*" or "posttraumatic stress" or "posttraumatic symptom*" or "traum* symptom*" or "traumatic stress" or "mood disorder*" or "affective disorder*" or "internali* symptom*" or "psychological distress" or "adult mental health" or "low mood").mp. (2440024)

4     1 and 2 and 3 (8064)

5     limit 4 to yr="1860 - 2010" (1964)

6     limit 4 to yr="2008 - 2015" (2980)

7     limit 4 to yr="2013 -Current" (5394)

8     5 or 6 or 7 (8053)

9     4 not 8 (11)

10     remove duplicates from 5 (1154)

11     remove duplicates from 6 (1673)

12     remove duplicates from 7 (2965)

13     10 or 11 or 12 (4509)

14     9 or 13 (4520)

15     remove duplicates from 14 (4511)

[mp=ti, ab, hw, tc, id, ot, tm, mh, tn, dm, mf, dv, kw, fx, dq, nm, kf, ox, px, rx, an, ui, sy
